# Veterinary Post Mortem Examinations

**Published:** 1891-08

**Authors:** 


VETERINARY POST-MORTEM EXAMINATIONS, by *A. W. Clement, V. S.* Published by Sabiston and Murray, New York.

Autopsies upon the lower animals are frequently made, but the reports thereon are often far from lucid. A lack of system is evident at first glance in many of them. A guide in making these post-mortem examinations based on a system of dissection which shall point out the anatomical variations among the animals and the differences between them and man, is what every Veterinarian needs and he will find it in this little book.

He will also find the best methods of recording the autopsies, and some cases by the author introduced to illustrate these methods and which are of themselves of much interest. The book contains many valuable illustrations.

